# Surgical and Endovascular Management of Pseudoaneurysms of the Ascending Aorta and Aortic Root: Operative Strategies and Long-Term Outcomes

**DOI:** 10.1055/a-2875-3047

**Published:** 2026-05-24

**Authors:** Rafat Abu Ghannam, Gal Aviel, Noa Oren, David Planer, Gabby Elbaz-Greener, Ori Wald, Amit Korach

**Affiliations:** 1 Department of Cardiothoracic Surgery 58884 Hadassah Medical CenterJerusalemIsrael; 2 Department of Cardiology 58884 Hadassah Medical CenterJerusalemIsrael

**Keywords:** aortic pseudoaneurysm, cardiac surgery, ascending aorta, endovascular repair

## Abstract

**Background:**

Ascending aortic and aortic root pseudoaneurysms are rare but potentially life-threatening complications, most commonly occurring after prior cardiac or aortic interventions. Management remains challenging and may require complex surgery or, in selected cases, endovascular therapy. We reported the experience of a single cardiovascular center in the management of aortic pseudoaneurysms.

**Methods:**

We retrospectively reviewed 12 consecutive patients treated for thoracic aortic pseudoaneurysms between August 2008 and November 2025. Demographic, perioperative, and follow-up data were obtained from the institutional Society of Thoracic Surgeons (STS) database. Descriptive statistics were used for baseline characteristics and outcomes. Overall survival was estimated using Kaplan–Meier analysis.

**Results:**

Mean patient age was 56 ± 14 years, and 83.3% were male. All patients had prior cardiac interventions. Five patients (41.6%) had infected pseudoaneurysms. Median pseudoaneurysm size was 6.55 cm. Ten patients underwent open surgical repair, and two underwent percutaneous treatment. Hospital mortality was 8.3%. Postoperative stroke occurred in 16.7%, acute kidney injury in 16.7%, and atrial fibrillation in 25%. Median follow-up was 28 months with overall mortality of 16.7%. Estimated survival at the end of follow-up was 85%. One late death occurred in a patient with candidemia and recurrent infection.

**Conclusion:**

Surgical repair of ascending aortic pseudoaneurysms can be performed with acceptable morbidity and mortality and is effective in most patients. Infected pseudoaneurysms remain particularly challenging. In carefully selected high-risk patients, endovascular approaches provide a feasible less-invasive alternative with reasonable mid-term outcomes.

## Introduction

Pseudoaneurysm of the ascending aorta and aortic root is a disruption of all layers of the aortic wall that does not result in fatal exsanguination. Aortic pseudoaneurysms typically arise from arterial injury, most commonly due to blunt or penetrating trauma, infection, dissection of the aorta, atherosclerotic disease, connective tissue disease, or iatrogenic causes as a result of cardiac or aortic invasive interventions. 1

Aortic pseudoaneurysms are uncommon and occur more frequently in middle-aged male patients. Their incidence varies according to etiology and location. A meta-analysis reported an incidence of 5.3 per 100,000 individuals per year, with a prevalence of 0.16%. These lesions carry a high risk of rupture, embolization, or compression of adjacent structures, contributing to substantial morbidity and mortality. 2

**Fig. 1:**
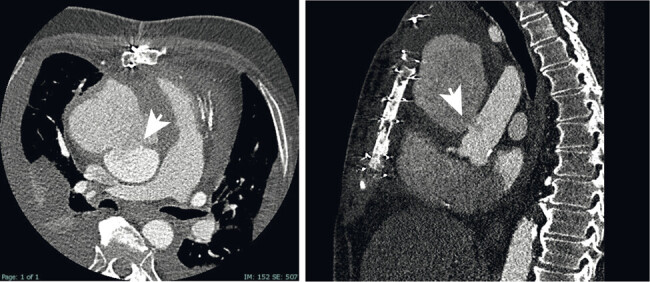
Computer tomography images of an aortic pseudoaneurysm. Axial (left) and sagittal (right) images, showing a communication (arrowhead) between the aortic lumen and a contained rupture.

Postoperative aortic pseudoaneurysms most often arise at sites of arterial cannulation or along anastomotic suture lines. In these settings, a limited defect in the vessel wall permits blood to escape into the adjacent tissues, gradually forming a contained sac ( Fig. 1 ). 3 Investigations aimed at defining predisposing factors have identified several contributors, including elevated hemodynamic stress at the anastomosis, compromised quality of the remaining aortic wall, progressive vascular pathology, infection, and technical aspects of the repair, such as suture technique and prosthetic material. 4

Most patients are asymptomatic, and pseudoaneurysms are often detected incidentally on chest radiography or computed tomography (CT). When symptoms do occur, chest pain is the most common presentation. Larger pseudoaneurysms may also cause dyspnea, dysphagia, or hoarseness due to mass effect, depending on their respective anatomical location. 5

The majority of cases necessitate complex surgical interventions. However, recent advances in endovascular techniques have provided less invasive alternatives to conventional open repair, particularly in carefully selected patients. The choice of intervention is based on the patient perioperative risk and the pseudoaneurysm's characteristics, including size, location, and morphology. 6 7 8 9

In this study, we present the experience from a single cardiovascular center of 12 cases of aortic pseudoaneurysm and discuss the treatment strategies employed.

## Materials and Methods

Between August 2008 and November 2025, 12 consecutive patients with aortic pseudoaneurysms were treated at our institution. The demographic, perioperative, imaging, and follow-up data were retrospectively reviewed using our departmental STS database. Patients with thoracic aortic pseudoaneurysms were included in the cohort, regardless of the etiology. Clinical follow-up was obtained as part of the routine follow-up protocols of our department.

We used descriptive statistics to analyze the baseline characteristics and clinical outcomes. To calculate overall survival, we generated a Kaplan–Meier survival curve using the *Survival* package in RStudio, Version 2023.09.1 + 494.

The study was approved by the Hadassah Hebrew University Medical Center Helsinki Committee. The need for informed consent was waived by both committees, since this was a retrospective study using unidentified data obtained from departmental databases.

## Results

### Patient Demographics and Presentation

There were 10 male (83.3%) and 2 female (16.7%) patients with a mean age of 56 ± 14 ( Table 1 ). All patients had undergone a prior cardiac intervention (11 cardiac surgery, 1 percutaneous intervention). One patient (8.3%) presented with cardiac tamponade. Five patients (41.6%) presented with an infected pseudoaneurysm, based on a typical imaging appearance and positive cultures ( Table 2 ). Blood cultures were positive for *Staphylococcus aureus* (3/5), *Candida albicans* (1/5), and unidentified gram-negative rods (1/5). The median pseudoaneurysm size was 6.55 cm (5.75–10.25) ( Table 3 ). Four patients required emergent intervention due to hemodynamic instability (33.3%), seven patients were treated urgently (58.3%), and one patient was admitted electively for intervention (8.3%). The time interval between previous cardiac procedure and the index intervention was classified as within 1 year of surgery (41.7%), between 1 and 5 years of surgery (25%), and more than 5 years (33.3%).

**Table 1:** Baseline characteristics and demographics

Variant	Value percentage
Age (y), mean ± SD	56 ± 14
BMI (kg/m ^2^ ), mean ± SD	28 ± 4.5
Male	10 (83.3%)
Comorbidities	
• Hypertension	10 (83.3%)
• Diabetes	5 (41.7%)
• Dyslipidemia	9 (75%)
• Smoking	4 (33.3%)
• COPD	0 (0%)
• Hemodialysis	1 (8.3%)
• Stroke	2 (16.7%)
• Coronary artery disease	6 (50%)
• Peripheral artery disease	1 (8.3%)
• Carotid artery disease	1 (8.3%)
• Steroidal therapy	1 (8.3%)
• Anticoagulation therapy	11 (91.6%)
• Prior MI	5 (41.7%)
Prior coronary intervention	3 (25%)
Prior surgical interventions	11 (91.6%)
• Prior CABG	2 (18%)
• Prior valve surgery	2 (18%)
• Prior aortic surgery	5 (45.4%)
• Combined procedure	2 (18%)
Initial presentation	
• Chest pain	6 (50%)
• Shortness of breath	2 (16.7%)
• Fever	2 (16.7%)
• Shock	2 (16.7%)
NYHA classification	
• Class 1	4 (33.3%)
• Class 2	5 (41.7%)
• Class 3	2 (16.7%)
• Class 4	1 (8.3%)
Case classification	
• Elective	1 (8.3%)
• Urgent	7 (58.3%)
• Emergent	4 (33.3%)
Management	
• Open surgery	10 (83.3%)
• Endovascular	2 (16.7%)

Abbreviations: BMI, body mass index; CABG, coronary artery bypass grafting; COPD, chronic obstructive pulmonary disease; MI, myocardial infarction; NYHA, New York Heart Association; SD, standard deviation.

**Table 2:** Clinical, operative, and outcome characteristics of the study cohort

Age	Sex	Surgical urgency	Previous intervention	Time interval to current intervention (mo)	Indication of intervention	Procedure	Leak site	Outcome
52	M	Urgent	Bentall	120.0	Infected pseudoaneurysm	Primary repair with pericardial patch	Distal aortic graft anastomosis	Expired
38	F	Urgent	Bentall	36.0	Pseudoaneurysm	Primary repair and coronary artery bypass grafting	Right coronary artery button	Alive
24	M	Urgent	AVR	2.0	Infected pseudoaneurysm	Mechanical Bentall and pulmonary trunk replacement	Aortotomy	Alive
51	M	Emergent	AVR	2.0	Pseudoaneurysm	Replacement of the ascending aorta with interposition graft	Cannulation site	Alive
62	M	Urgent	Bentall	24.0	Pseudoaneurysm	Bio-Bentall and right atrium wall repair	Noncoronary cusp	Alive
50	M	Emergent	Coronary angiography	2.0	Aortic rupture	Replacement of the ascending aorta with interposition graft	Aortic root	Alive
45	M	Urgent	Bentall	12.0	Infected pseudoaneurysm	Bio-Bentall	Multiple leak sites	Expired
80	F	Urgent	Replacement of ascending aorta with interposition graft, AVR, MV annuloplasty, and CABG	108.0	Infected pseudoaneurysm	Redo CABG and replacement of the ascending aorta with interposition graft	Proximal coronary graft	Alive
50	M	Emergent	Replacement of ascending aorta with interposition graft and AVR	24.0	Pseudoaneurysm	Primary repair of pseudoaneurysm	Proximal aortic graft anastamosis	Alive
59	M	Emergent	CABG	12.0	Infected pseudoaneurysm	Stent graft GORE TAG 40/100	Proximal coronary graft	Alive
77	M	Urgent	CABG	144.0	Pseudoaneurysm	Nexus stent graft	Aortic cannulation site	Alive
64	M	Elective	Bentall	120.0	Pseudoaneurysm	Primary repair of pseudoaneurysm and reanastomosis of the right coronary button	Distal aortic graft suture line and right coronary button	Alive

Abbreviations: AVR, aortic valve replacement; CABG, coronary artery bypass grafting.

**Table 3:** Operative-specific characteristics

Number of previous surgeries, median [IQR]	1 [1–2]
Previous procedure	
• PCI	1 (8.3%)
• Isolated CABG	2 (16.7%)
• Aortic root	5 (41.6%)
• Isolated AVR	2 (16.7%)
• Combined procedure	2 (16.7%)
Pseudoaneurysm size (cm), median [IQR]	6.55 [5.75–10.25]
Operation interval (mo), median [IQR]	24 [4.5–117]
• ≤1 y	5 (41.7%)
• 1–5 y	3 (25%)
• ≥5 y	4 (33.3%)
Cardio-pulmonary bypass time (min), median [IQR]	150 [115–218.5]
Aortic Cross clamp time (min), median [IQR]	86 [40.5–174.5]
Circulatory arrest, median (min), median [IQR]	11 [6.5–20]
Hypothermia (°C), median [IQR]	28 [20–30.4]
Site of leak	
• Proximal anastomosis of aortic graft	1 (8.3%)
• Distal anastomosis of aortic graft	1 (8.3%)
• Coronary graft	3 (25%)
• Aortotomy	1 (8.3%)
• Aortic root	2 (16.7%)
• Cannulation site	2 (16.7%)
• Multiple leak sites	2 (16.7%)
Repair Method	
• Primary repair	4 (33.3%)
• Ascending aorta replacement	3 (25%)
• Bentall	2 (16.7%)
• Bentall with pulmonary trunk replacement	1 (8.3%)
• Stent graft	2 (16.7%)

Abbreviations: VR, aortic valve replacement; CABG, coronary artery bypass grafting; IQR, interquartile range; PCI, percutaneous coronary intervention.

### Operative Intervention

The operative strategy in all patients who underwent surgery was femoral arterial and venous cannulation for conduction of cardiopulmonary bypass, medium hypothermia (28–32°C), followed by redo median sternotomy and total circulatory arrest. Following exploration of the ascending aorta, a cross-clamp was placed on this vessel, and cardiopulmonary bypass was reinstituted. The median cardiopulmonary bypass time was 150 minutes (115–218.5), median cross clamp-time was 86 minutes (40.5–174.5), and the median circulatory arrest time was 11 minutes (6.5–20; Table 3 ).

The operative method of repair depended on the specific aortic pathology. The most frequent leak sites were noted to be proximal anastomosis site of a coronary graft (25%) followed by prior cannulation site or multiple leak sites (16.6% each). Replacement of the ascending aorta with an interposition graft was performed in 3/10 patients (30%), and a Bentall procedure was undertaken in 2/10 patients (20%). A primary repair of the leak site was employed in 4/10 patients (40%; Tables 2 and 3 ).

### Percutaneous Repair

Two patients underwent a percutaneous repair. One patient developed a pseudoaneurysm after a previous coronary artery bypass grafting (CABG), with subsequent mediastinal bleeding and mediastinitis. Due to hemodynamic instability with cardiogenic shock, it was decided to repair it percutaneously. The closure of the aortic leak using a covered stent graft was confirmed in angiography and transesophageal echocardiography. Residual mediastinal hematoma was evacuated surgically 7 days later. Significant oozing from the aortic wall was controlled by implanting a bovine pericardial patch mounted with biological glue.

The second patient likewise presented after a prior CABG. As the leak originated from the previous aortic cannulation site, NEXUS DUO Aortic Arch Stent Graft System (Endospan Ltd.) was employed after a left subclavian to left carotid bypass was performed.

### Early Follow-up

There was one hospital death (8.3%) due to postoperative low cardiac output syndrome in a patient with preoperative poor left ventricular function ( Table 4 ). The median hospital stay was 21 days (8–30). Four patients (33.3%) were extubated >48 hours after the procedure. Two patients (16.7%) were diagnosed with postintervention cerebrovascular accident (one in the surgical arm and one in the percutaneous arm). Both patients were discharged home without neurological sequelae. One patient that underwent aortic and pulmonary root replacement due to infected pseudoaneurysm developed a high-grade fever on postoperative day 10. CT demonstrated a perigraft collection, prompting reexploration of the chest. Mediastinitis was confirmed, with cultures yielding *Klebsiella pneumoniae* . The patient was managed with local and systemic antibiotic therapy, following clinical recovery and the absence of any residual signs of infection, and was subsequently discharged home. Two patients (16.7%) developed acute kidney injury, which did not require renal replacement therapy. Postintervention atrial fibrillation occurred in three patients (25%). There were no deep sternal wound infections.

**Table 4:** Early postoperative complications

Hospital length of stay (d), median [IQR]	21 [8–30]
In-hospital mortality	1 (8.3%)
AF	3 (25%)
Stroke	2 (16.7%)
AKI	2 (16.7%)
Prolonged intubation	4 (33.3%)
Infection	3 (25%)
• Mediastinitis	1(8.3%)
• Sepsis	1 (8.3%)
Reexploration	2 (16.7%)
Perioperative MI	0 (0%)
Packed red blood cell units transfused, median [IQR]	2 [1–7.5]

Abbreviations: AF, atrial fibrillation; AKI, acute kidney injury; IQR, interquartile range; LOS, length of stay; MI, myocardial infarction.

### Late Follow-up

**Fig. 2:**
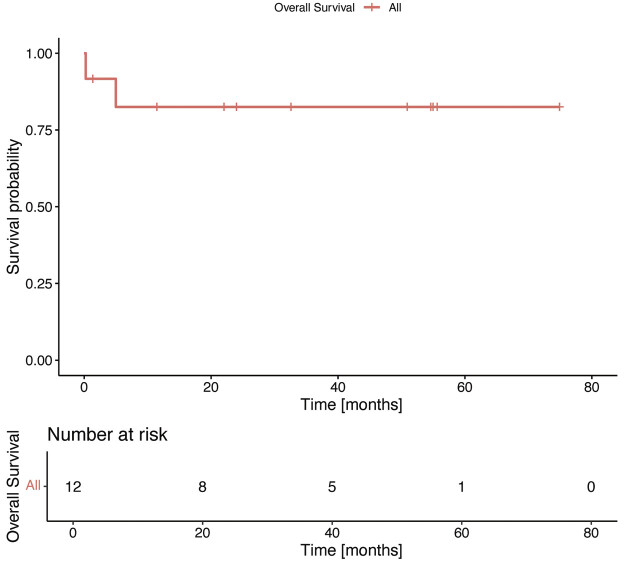
Survival curve. Kaplan–Meier analysis of overall survival showing an estimated 85% survival probability at last follow-up. Numbers of patients at risk are displayed beneath the curve.

The median follow-up time was 28 months (8–54; Table 5 ). Long-term follow-up was available in 10/12 patients (83%). The overall mortality was 16.7% ( *n* = 2), and the survival probability at the end of follow-up time was 85%, as shown in the Kaplan–Meier survival plot ( Fig. 2 ). A patient with candidemia and an infected pseudoaneurysm experienced recurrence and died 5 months after the initial repair during reoperation at another hospital. One patient required reintervention for aortic root dilatation 41 months after the operation. On clinical follow-up, two patients reported a New York Heart Association (NYHA) functional class of I (17%), 5 patients had an NYHA II (42%), and 1 patient reported NYHA III (8%).

**Table 5:** Late follow-up

Overall mortality	2 (16.7%)
Follow-up time, d (IQR)	862.25 (250.5–1671.5)
Reoperation	1 (8.3%)
NYHA (± SD)	2 (± 0.53)

Abbreviations: IQR, interquartile range; NYHA, New York Heart Association; SD, standard deviation.

## Discussion

Aortic pseudoaneurysm is a heterogenous disease and its presentation depends on the precise location and etiology of the lesion. 9 Consistent with other series, in our report, the most frequent presenting symptoms were chest pain and dyspnea, accounting for 75% of presenting complaints. 7 8 These symptoms arise from local mass effect on adjacent mediastinal organs or from concomitant aortic valve insufficiency with subsequent development of heart failure. However, it is important to bear in mind that a minority of patients presents with catastrophic aortic events such as aortic rupture. Such presentation mandates an emergent surgical intervention, which is almost uniformly a redo-sternotomy, with its additive surgical complexity. Due to the small size of most case series reporting the clinical outcome of patients with aortic pseudoaneurysms, it is difficult to analyze a possible interaction between the presenting symptom and clinical outcomes and mortality.

The surgical repair strategy implemented depends on the precise lesion location (i.e., the leak site), its relation to the coronary arteries and adjacent cardiac chambers, and additional comorbidities such as the presence of mediastinitis or concomitant aortic valve pathology. From our experience, most pseudoaneurysms can be managed by primary repair or replacement of the ascending aorta using an interposition graft. These rather straightforward approaches were undertaken successfully in 58% of our patients in the surgical arm. However, consistent with previous reports, the surgical complexity increases if the lesions involve the coronary ostia or in the presence of an infected pseudoaneurysm, which mandates radical tissue debridement. 2 7 8

The presence of occult or overt mediastinal infection, especially in the context of prior aortic replacement with a graft, is an important contributor to the disruption of aortic layers and the development of a pseudoaneurysm. Some series report an approximately 50% prevalence of associated infection, with only a minority of patients present with positive blood cultures. 7 A common isolated pathogen is *Salmonella* species. 8 In our series, five patients were operated with known positive blood cultures for *Staphylococcus aureus, Gram-negative rods* , *and Candida albicans.* These patients were later found to have infected grafts on subsequent pathological and bacteriological examinations of the surgical specimens. The differences in pathogens between our series and others probably reflect different referral patterns and population-associated characteristics.

Endovascular therapy for the treatment of ascending aortic pathology has remained limited, reflecting the limited landing zone for stent deployment, and proximity to vital structures such as the aortic valve, coronary ostia, and neck vessels. In this series, we describe two patients with ascending aortic pseudoaneurysms that were managed percutaneously due to prohibitive surgical risk. One of the patients had active mediastinitis, harboring an increased risk for chronic infection due to the lack of radical tissue debridement as is possible in an open surgical approach. However, several case reports have shown favorable outcomes, offering palliation in this sick patient population. 6 10 11

This study is not without limitations. This is a small retrospective case series from a single center, and selection bias in terms of referral policy and population characteristics are unavoidable. Additionally, we do not report data on aortic pseudoaneurysms of the descending aorta, which behave differently and are usually the results of trauma to a penetrating atherosclerotic ulcer. 9

## Conclusion

Surgical treatment of ascending aortic pseudoaneurysms is associated with favorable outcomes and can usually be achieved using limited reconstructive approaches. Patients with infected pseudoaneurysms pose a special challenge due to active infection and the need for extensive debridement. However, in carefully selected patients, endovascular treatment is a viable option, which can achieve reasonable palliation.

## References

[1] Writing Committee Members Isselbacher E M Preventza O Hamilton Black Iii J 2022 ACC/AHA Guideline for the Diagnosis and Management of Aortic Disease: a report of the American Heart Association/American College of Cardiology Joint Committee on Clinical Practice Guidelines J Am Coll Cardiol 2022 80 24 e223 e393 36334952 10.1016/j.jacc.2022.08.004 PMC9860464

[2] Gouveia E Melo R Silva Duarte G Lopes A Incidence and prevalence of thoracic aortic aneurysms: a systematic review and meta-analysis of population-based studies Semin Thorac Cardiovasc Surg 2022 34 01 1 16 33705940 10.1053/j.semtcvs.2021.02.029

[3] Rivera P A Dattilo J B Pseudoaneurysm. StatPearls [Internet] StatPearls Publishing 2024 31194401

[4] Kouchoukos N T Dougenis D Surgery of the thoracic aorta N Engl J Med 1997 336 26 1876 1888 9197217 10.1056/NEJM199706263362606

[5] Sullivan K L Steiner R M Smullens S N Griska L Meister S G Pseudoaneurysm of the ascending aorta following cardiac surgery Chest 1988 93 01 138 143 3257182 10.1378/chest.93.1.138

[6] Quevedo H C Alonso A Endovascular therapy for ascending aorta pseudoaneurysm Cardiovasc Revasc Med 2016 17 08 586 588 27640128 10.1016/j.carrev.2016.08.008

[7] Atik F A Navia J L Svensson L G Surgical treatment of pseudoaneurysm of the thoracic aorta J Thorac Cardiovasc Surg 2006 132 02 379 385 16872966 10.1016/j.jtcvs.2006.03.052

[8] Ting A C Cheng S W Ho P Poon J T Tsu J H Surgical treatment of infected aneurysms and pseudoaneurysms of the thoracic and abdominal aorta Am J Surg 2005 189 02 150 154 15720981 10.1016/j.amjsurg.2004.03.020

[9] Manenti A Roncati L Sorrentino L Thoracic aortic pseudoaneurysm: Inside its pathophysiology Vascular 2025 33 04 821 828 39118321 10.1177/17085381241273314

[10] Liu C-W Ye W Liu B Zeng R Wu W Dake M D Endovascular treatment of aortic pseudoaneurysm in Behçet disease J Vasc Surg 2009 50 05 1025 1030 19660895 10.1016/j.jvs.2009.06.009

[11] Basu R Zhang J Zaheer S Grimm J Szeto W Kalapatapu V Ascending aorta thoracic endovascular aortic repair for infected pseudoaneurysm J Vasc Surg Cases Innov Tech 2022 8 02 244 247 35510219 10.1016/j.jvscit.2022.02.005 PMC9058959

